# The Role of Family-Centred Approaches in Promoting Inclusive Mental Health Care for Individuals with Psychiatric Disorders: A Scoping Review

**DOI:** 10.3390/ijerph23080952

**Published:** 2026-07-24

**Authors:** Leshata Winter Mokhwelepa, Gsakani Olivia Sumbane

**Affiliations:** School of Medicine, Faculty of Health Science, University of Limpopo, Private Bag X 1106, Sovenga, Polokwane 0727, South Africa; gsakani.sumbane@ul.ac.za

**Keywords:** family-centred care, mental health, inclusivity, psychiatric disorders, scoping review

## Abstract

**Highlights:**

**Public health relevance—How does this work relate to a public health issue?**

- Addresses the widespread public health challenge of mental health disorders through family involvement.
- Promotes community-based, inclusive mental health care to improve access and reduce stigma.

**Public health significance—Why is this work of significance to public health?**

- Highlights the role of family-centred approaches in enhancing treatment outcomes and social inclusion.
- Supports efforts to reduce disparities and promote mental health equity at the population level.

**Public health implications—What are the key implications or messages for practitioners, policy makers and/or researchers in public health?**

- Encourages practitioners to adopt family-centred strategies for better patient engagement.
- Informs policymakers to create supportive policies and allocate resources for inclusive mental health services.

**Abstract:**

Family-centred approaches are being increasingly recognized as integral to inclusive mental health care. Although substantial evidence-based exists for specific interventions particularly family intervention for psychosis, there is no synthesis that maps how family-centred approaches are conceptualized, implemented, and linked to inclusivity across diagnoses, service contexts, and levels of evidence. Existing reviews are largely disorder-specific or outcome-focused and do not examine family-centred care as a system-level strategy for inclusive practice. This scoping review aimed to map and synthesize the existing literature on family-centred approaches used to promote inclusive mental health care for individuals with psychiatric disorders. This review followed the Joanna Briggs Institute (JBI) methodology for scoping reviews and the PRISMA-ScR reporting guidelines. Searches were conducted in PubMed, PsycINFO, CINAHL, Scopus, Embase, and Web of Science for English-language empirical studies published between 2000 and 2025. Studies involving individuals with psychiatric disorders and any form of family-centred, family-focused, or family-inclusive approach were eligible. Data were charted and analyzed thematically to map intervention types, reported outcomes, and implementation influences; the review did not evaluate intervention effectiveness. Twenty-four studies met the inclusion criteria. Only five themes emerged from this study. Across the literature, family involvement was associated with improved engagement, enhanced communication, and increased caregiver competence. Psychoeducation featured prominently in evidence-based interventions, while newer models emphasized shared decision-making and service co-production. Family-centred approaches are conceptualized in diverse ways across mental health services and are commonly linked to more inclusive and collaborative care. However, the strength of evidence varies substantially between intervention types. This review clarified how family-centred practices are situated across levels of evidence and service contexts, highlighting the need for greater precision in distinguishing established interventions from emerging practices and for system-level strategies to support consistent, culturally responsive family inclusion.

## 1. Introduction

Inclusivity in mental health care refers to the delivery of services that are equitable, accessible, and culturally responsive, ensuring that individuals receive appropriate care regardless of their social or demographic background [1]. The World Health Organization (WHO) identifies families as central contributors to social support, daily functioning, and long-term outcomes for individuals living with psychiatric disorders [2].

The importance of family involvement in inclusive mental health care can be understood through several interrelated mechanisms. Families often act as a bridge between patients and services by facilitating communication, supporting help-seeking, and ensuring continuity of care across clinical and community settings. They also reduce social isolation by providing ongoing emotional and practical support, which is particularly important for individuals who may withdraw due to symptoms or stigma. In addition, families can support reintegration into the community by assisting with daily functioning, rehabilitation, and social participation. Importantly, family involvement may also serve as a protective factor against stigma by reinforcing acceptance, normalization of mental illness within the home environment, and buffering individuals from negative societal attitudes [3].

Recent research consistently demonstrates that family-centred interventions in mental health care are associated with significant positive clinical outcomes, particularly in individuals with severe psychiatric disorders such as schizophrenia [3]. Evidence from systematic reviews and meta-analyses indicates that involving family members in treatment contributes to reduced relapse rates, improved medication adherence, and decreased hospital readmissions compared to standard care alone [3,4]. These outcomes are largely attributed to enhanced psychoeducation, improved communication within family systems, and the creation of supportive home environments that facilitate treatment continuity and early identification of symptom exacerbation.

Despite this established evidence base, the way “family-centred care” is conceptualized and operationalized across mental health systems remains highly variable. Existing reviews predominantly focus on discrete diagnostic groups (e.g., schizophrenia or bipolar disorder) or evaluate the effectiveness of specific interventions such as psychoeducation or behavioural family therapy [5,6]. What remains unclear is how family-centred approaches are being applied more broadly as a strategy for inclusive mental health care across diagnoses, service settings, and cultural contexts. In other words, while we know that family interventions are effective for specific disorders, we lack a synthesis that maps how family-centred practices are conceptualized, implemented, and linked to inclusivity within contemporary mental health systems.

Historically, psychiatric services have prioritized individualized, biomedical models of care, often positioning families as peripheral or problematic. This orientation has limited opportunities for families to participate meaningfully in assessment, planning, and recovery processes. Research has shown that exclusion of families may increase caregiver burden, exacerbate stigma, and undermine continuity of care [7,8,9,10]. Conversely, service models such as Open Dialogue illustrate how structured family involvement can reshape care trajectories, with reported benefits for engagement, satisfaction, and long-term functioning [11]. These developments raise an open empirical question: how are family-centred approaches currently being used to promote inclusive mental health care, beyond disorder-specific intervention frameworks?

Inclusivity in mental health care also requires attention to the cultural and structural contexts in which families provide support. In many low- and middle-income countries (LMICs), families function as primary caregivers due to limited formal services [12]. In Indigenous, migrant, and minority communities, culturally responsive family involvement has been linked to improved engagement and trust in services [13,14]. However, stigma, discrimination, and social risk continue to shape whether families can participate openly in care [15]. Understanding how family-centred approaches are framed and implemented across diverse contexts is therefore essential for translating evidence-based family work into inclusive systems of care.

Family-centred models also recognize the reciprocal relationship between service-user recovery and family wellbeing. Caregivers frequently experience emotional distress, financial strain, and role overload [16,17]. Evidence-based family interventions, including Cognitive Behavioural Family Interventions (CBFIs), have been shown to reduce expressed emotion, a well-established predictor of relapse in schizophrenia [18,19]. These interventions have also been associated with improvements in both patient and caregiver outcomes, including symptom management, medication adherence, and reduced caregiver burden [19,20,21]. However, newer family-inclusive practices are often described in policy and service literature without equivalent empirical grounding. Without clear differentiation, there is a risk that emerging or loosely defined practices may be implicitly legitimized through association with rigorously tested interventions. Therefore, a scoping review is appropriate to map how family-centred approaches are defined, described, and applied within mental health care, with a focus on their role in inclusive service delivery for individuals with psychiatric disorders.

### Study Aim

This scoping review aimed to map and synthesize the existing literature on family-centred approaches used to promote inclusive mental health care for individuals with psychiatric disorders.

## 2. Methodology

### 2.1. Study Design

This scoping review was conducted in accordance with the Joanna Briggs Institute (JBI) methodological guidance for scoping reviews [22] and is reported in line with the PRISMA-ScR (Supplementary Materials Table S1) framework [23]. This study directly responds to the identified gap in the literature, where existing reviews focus on disorder-specific interventions rather than family-centred care as a system-level, inclusive practice. The review followed the five JBI stages:

1. Identifying the research question;
2. Identifying relevant studies;
3. Study selection;
4. Charting the data;
5. Collating, summarizing, and reporting the results.

#### 2.1.1. Identifying the Research Question

The primary research question guiding this review was:

What is the role of family-centred approaches in promoting inclusive mental health care for individuals with psychiatric disorders?

Sub-questions included:

- *What types of family-centred interventions are used in the management of psychiatric disorders?*
- *How are family members involved in the care, support, and recovery of individuals with psychiatric disorders?*
- *What barriers and facilitators influence the implementation of family-centred approaches in mental health services?*

##### Eligibility Criteria

Eligibility criteria were defined according to JBI guidance and aligned with the review’s PCC framework shown in Table 1 below.

#### 2.1.2. Identifying Relevant Studies

The literature search was initiated in January 2025 and concluded in December 2025. The authors of this study only reviewed studies published in English. To enhance the precision and comprehensiveness of the search, a health sciences librarian with expertise in systematic and scoping review methodology collaborated in the development and refinement of the search strategy. This review focused on studies published in English to ensure accurate interpretation and synthesis of the evidence. Given resource and time constraints, including only English-language publications allowed for a rigorous and consistent analysis, while maintaining methodological quality and clarity in reporting. The inclusion of a librarian is reported to demonstrate methodological transparency and adherence to best practices, as recommended by JBI and PRISMA guidance. Publications were limited to the period of 2000–2025. This timeframe was selected to reflect the emergence of contemporary recovery-oriented and person-centred mental health frameworks, within which family-centred care has been most actively theorized and implemented. Earlier foundational family therapy literature predates current models of inclusive and recovery-oriented practice and was therefore considered conceptually distinct from the focus of this review. Searches were conducted in PubMed, CINAHL, PsycINFO, Scopus, Embase, and Web of Science. Controlled vocabulary (e.g., MeSH terms) and free-text keywords were combined using Boolean operators. Search terms included variations of:

- *Family-centred care, family-focused practice, family-inclusive, caregiver involvement, family intervention;*
- *Mental disorders, psychiatric disorder, serious mental illness, psychosis, bipolar disorder, depression*

Grey literature sources were also searched to reduce publication bias, and reference lists of included studies were manually screened to identify additional eligible articles. The full search strategy is shown below in Table 2.

#### 2.1.3. Study Selection

The database search yielded 3620 records. After the removal of 1145 duplicates, 2475 unique citations remained. Two independent reviewers screened titles and abstracts against the eligibility criteria. At this stage, 2310 records were excluded because they did not involve psychiatric populations, did not include a family-centred component, or were not empirical studies. Full texts of 165 articles were sought for retrieval. Of these, 91 articles could not be retrieved despite multiple strategies, including institutional access, interlibrary loan requests, and direct author contact where necessary. The remaining 74 full-text articles were retrieved and assessed independently by both reviewers against the eligibility criteria.

**Figure 1:**
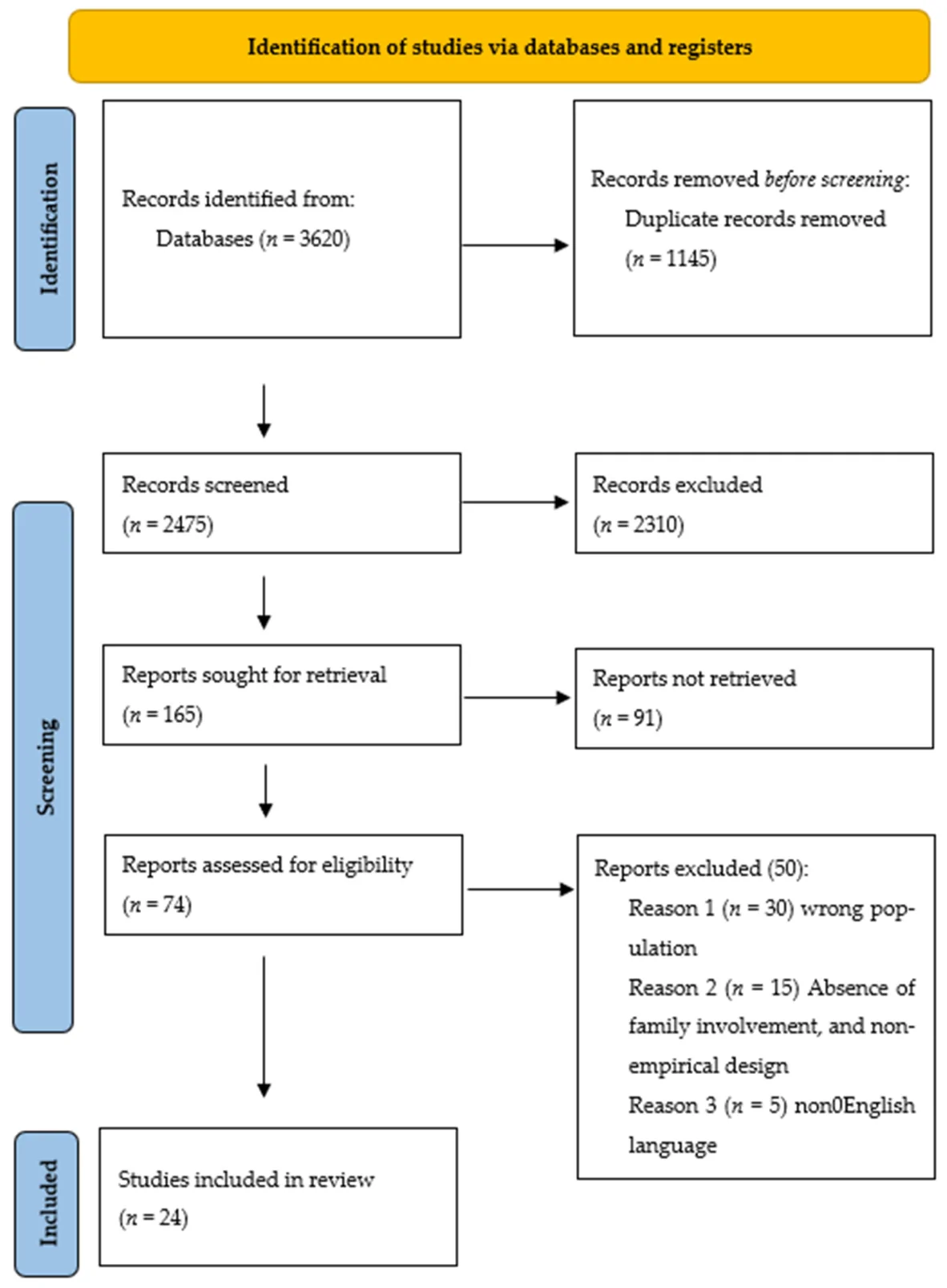
PRISMA flow diagram showing the study selection [23].

At the full-text screening stage, 50 articles were excluded for reasons including wrong population, absence of family involvement, non-empirical design, and non-English language. Ultimately, 24 studies met all inclusion criteria and were included in the final review. Any disagreements between reviewers were resolved through discussion, and a third reviewer cross-checked a subset of decisions to ensure consistency and auditability. All screening decisions were documented to maintain transparency and reproducibility. The study selection process is summarized in the PRISMA flow diagram (Figure 1).

It is important to note that we have made multiple efforts to obtain full-text versions of all potentially eligible articles. First, we accessed articles via institutional subscriptions to all relevant databases (PubMed, CINAHL, PsycINFO, Scopus, Embase, Web of Science). For articles not available through these databases, we contacted corresponding authors directly via email to request copies. Additionally, interlibrary loan services were used for journals not subscribed to by our institution. This limitation has been clearly reported in the PRISMA flow diagram and acknowledged as a potential source of missing data in the review.

During study selection, articles were not included based solely on the presence of family involvement. Instead, studies were screened for explicit reference to family-centred practices in relation to service user engagement, participation, or person-centred care. Where inclusivity was not explicitly stated, studies were included only if sufficient contextual evidence indicated that family involvement was embedded within collaborative, supportive, or recovery-oriented care processes.

Analysis

Thematic synthesis was conducted using an iterative approach following Braun and Clarke’s thematic analysis framework [24]. Initial codes were generated inductively from the extracted data, with no pre-existing coding framework applied at the outset. However, the development of themes was guided by the study objectives and review questions, allowing for a hybrid inductive–deductive approach. Two reviewers independently coded the extracted findings. Codes were compared and discrepancies were resolved through discussion and consensus. Where disagreement persisted, a third reviewer was consulted. Codes were then grouped into categories and refined into overarching themes through iterative discussion and constant comparison across studies. The final themes were reviewed against the dataset to ensure internal coherence and external distinction. Table 3 shows the summary of the thematic synthesis.

#### 2.1.4. Charting the Data

A data extraction form was developed in line with JBI guidance and piloted on a subset of studies. Data from the 24 included studies were charted using a structured extraction form developed in line with JBI guidance and piloted on a subset of studies to ensure consistency. The form captured key study characteristics, including author(s), year, country, study design, population, type of family-centred approach, and reported outcomes related to inclusivity, engagement, and implementation. Contextual notes were also recorded to capture cultural considerations, service settings, and reported implementation challenges. A summary of the characteristics of the studies is shown in Table 4.

During data extraction, information relating to inclusivity was captured based on how studies described service user involvement, shared decision-making, cultural responsiveness, and the role of families in care processes. Where “inclusivity” was not explicitly defined in the included studies, it was interpreted based on the authors’ descriptions of engagement practices while maintaining a distinction between general family involvement and practices that actively promoted inclusive care.

Analysis

## 3. Collating, Summarizing, and Reporting the Results

A total of 24 studies met the inclusion criteria and were included in this review. The studies were published between 2001 and 2024 and originated primarily from high-income countries, including the United Kingdom, United States, Australia, Canada, and Western Europe. The included studies comprised randomized controlled trials (*n* = 8), qualitative studies (*n* = 7), cross-sectional studies (*n* = 5), prospective/cohort/longitudinal studies (*n* = 3), and one service evaluation or study with an unclear design (*n* = 1).

This classification is used to inform the interpretation of findings and to distinguish between evidence derived from controlled intervention studies and that derived from descriptive or qualitative research. The conclusions drawn in this review are supported by both controlled intervention studies (randomized controlled trials) and descriptive evidence, including qualitative, cross-sectional, and cohort studies; specifically, conclusions regarding effectiveness of interventions are primarily based on controlled trials, while conclusions relating to experiences, associations, and contextual factors are mainly supported by descriptive and qualitative evidence.

The psychiatric populations represented included individuals diagnosed with psychotic disorders, bipolar disorder, major depressive disorder, personality disorders, and mixed severe mental illness cohorts. Family members included parents, partners, siblings, and informal caregivers. Importantly, the evidentiary strength of the included studies varied substantially. A subset evaluated well-established family interventions (e.g., structured family intervention for psychosis), while others described emerging or exploratory family-inclusive practices without formal outcome evaluation. These categories were analytically distinguished throughout synthesis to avoid conflation of evidence. Five themes surface from this study.

### 3.1. Theme 1: Enhancement of Treatment Engagement Through Collaborative Family Involvement

This theme captures evidence that family involvement plays a significant role in improving engagement with mental health services. Across studies, collaboration between families, service users, and clinicians was associated with improved treatment adherence, continuity of care, and sustained participation in services. Families often acted as facilitators of help-seeking and as intermediaries between service users and health systems, particularly in contexts where symptom severity or stigma limited autonomous engagement. However, the strength of evidence varied, with controlled intervention studies demonstrating more consistent improvements in engagement outcomes, while observational and qualitative studies emphasized the relational and contextual mechanisms underpinning these effects.

Across the reviewed literature, authors frequently report associations between family-centred approaches and improved treatment engagement among individuals with psychiatric disorders. Studies describing both structured interventions and routine service practices suggested that when families were invited into assessment sessions or care planning meetings, service users were more likely to attend scheduled appointments and to remain engaged with services [25,26,27,28,29,30]. For example, one study reported higher retention in outpatient programmes among young adults with first-episode psychosis when parents were involved in early consultations [31]. Another described improved medication adherence among individuals with bipolar disorder following the inclusion of family members in discussions about treatment goals and relapse warning signs [32].

It is important to note that these findings reflect the outcomes reported by study authors and vary in evidentiary strength. While some derive from controlled intervention trials, others emerge from observational or qualitative accounts.

### 3.2. Theme 2: Psychoeducation and Capacity-Building for Families

This theme highlights psychoeducation as a central mechanism through which family-centred care is operationalized. Studies consistently show that providing families with structured information about mental illness, treatment options, and relapse prevention improves caregiver confidence, communication, and problem-solving abilities. Capacity-building approaches also contribute to early identification of symptom relapses and improved coordination of care. While intervention studies demonstrate measurable improvements in patient and caregiver outcomes, qualitative evidence further illustrates how psychoeducation enhances caregivers’ sense of empowerment and reduces uncertainty in managing complex mental health conditions.

Studies reported improvements in families’ understanding of mental illness and confidence in their caregiving role following participation in structured educational sessions [32,33]. These sessions typically focused on symptom recognition, medication effects, communication strategies, and crisis management [31,32,33]. One study involving families of adolescents with mood disorders described parents feeling more capable of responding to emotional outbursts after attending a series of informational workshops led by mental health professionals [33]. Across the literature, psychoeducation is positioned as a mechanism through which families acquire practical knowledge and emotional readiness. However, while structured psychoeducational programmes are supported by stronger empirical traditions in some diagnostic areas, other accounts rely on experiential reports. This reinforces the need to distinguish between evidence-based intervention models and emerging family support practices when interpreting reported benefits.

### 3.3. Theme 3: Cultural, Social, and Structural Influences on Family Involvement

This theme reflects the contextual factors shaping the extent and nature of family participation in care. Cultural norms regarding collectivism, family responsibility, and interdependence often facilitate stronger family involvement, particularly in non-Western settings. However, social stigma, discrimination, and fear of judgement frequently limit open engagement with services. Structural factors such as service accessibility, resource constraints, and health care system capacity further influence the degree to which families can participate meaningfully in care processes. Collectively, these findings indicate that family-centred care is not universally implemented in the same way but is deeply embedded within broader sociocultural and systemic contexts.

Family involvement in mental health care was consistently shaped by cultural norms, social expectations, and structural conditions. In studies conducted within collectivist contexts, where family interdependence is culturally valued, multiple relatives were reported to attend appointments and to view themselves as central to the treatment journey [34,35,36,37]. By contrast, studies conducted in settings marked by high levels of mental health stigma described family hesitation, with relatives expressing concern about community judgement or social exposure [36,37,38].

These findings illustrate that family-centred care is not culturally neutral. Rather, its enactment is mediated by social meaning, stigma, and service accessibility. Family involvement therefore varies not only by clinical need but by the cultural and structural contexts within which services operate.

### 3.4. Theme 4: Barriers to Implementing Inclusive Family-Centred Care

This theme identifies persistent barriers that hinder the consistent implementation of family-centred approaches across mental health services. Key challenges include limited clinician training in family engagement, time constraints within clinical practice, unclear role definitions, and confidentiality concerns. Organizational limitations, including lack of structured family involvement protocols and insufficient resources, further restrict implementation. These barriers contribute to variability in practice, resulting in family involvement being dependent on individual clinician discretion rather than systematic service design. The evidence suggests that despite strong policy endorsement, operationalisation of family-centred care remains inconsistent.

Despite the perceived value of family-centred approaches, multiple barriers constrained their implementation. Several studies reported that clinicians lacked training in family-inclusive practices, resulting in inconsistent or superficial engagement [39]. Professional uncertainty around confidentiality, particularly when service users were reluctant to share information, further inhibited practice [40,41,42,43]. Time constraints were repeatedly identified, with high caseloads limiting opportunities for extended family meetings or follow-up [44,45]. Organizational systems often lacked dedicated pathways, spaces, or policies to support routine family involvement. Families themselves also encountered challenges, including emotional fatigue, uncertainty about their role, and fear of “interfering” with professional care [45].

These findings indicate that barriers operate at multiple levels—individual, professional, and systemic—suggesting that family-centred care cannot be sustained through clinician goodwill alone but requires structural and organizational support.

### 3.5. Theme 5: Benefits to Family Wellbeing and Reduction of Caregiver Burden

This theme focuses on the impact of family-centred interventions on caregiver outcomes. Evidence from both intervention and observational studies indicates that structured family involvement is associated with reduced caregiver burden, improved emotional wellbeing, and enhanced coping capacity. Psychoeducational and family-focused interventions also contribute to reductions in expressed emotion and improved understanding of mental illness within families. However, the literature also acknowledges that caregiving can remain emotionally and financially demanding, particularly in settings with limited formal support systems. These findings suggest that while family-centred care can improve caregiver outcomes, its effectiveness is influenced by both intervention design and contextual constraints.

Across studies, authors reported that family-centred approaches were associated with perceived improvements in family wellbeing and reductions in caregiver burden. Relatives described lower stress levels and emotional strain when they received structured support or were meaningfully included in care processes [45,46,47].

For example, caregivers of individuals with chronic psychosis reported feeling less overwhelmed after participating in family support groups that enabled shared reflection and peer connection [48]. Parents of adolescents with severe depression were described as developing improved coping strategies following family-based interventions focused on communication and emotional regulation [44,45].

While the strength of evidence varies, these findings consistently position family-centred care as addressing not only the needs of the service user but also the psychosocial wellbeing of caregivers. The literature thus frames families as both contributors to care and recipients of support, challenging models that treat them solely as adjuncts to treatment.

## 4. Discussion

Family-centred approaches are widely portrayed in the literature as enhancing treatment engagement among individuals with psychiatric disorders. Across studies, authors report that involving relatives in assessment, care planning, and follow-up is associated with improved adherence, reduced dropouts, and more stable participation in services [49,50]. Evidence from early psychosis research describes higher treatment retention when families assist with symptom monitoring or practical needs such as scheduling and transportation [28]. It is important to note, however, that these findings represent a mixture of empirically evaluated intervention effects and observational or experiential accounts. Collectively, the literature suggests that family involvement is perceived to strengthen therapeutic alliances and continuity of care, but the strength of the evidence varies substantially across contexts and diagnostic groups.

This review highlights a conceptual ambiguity in the literature on family-centred care. Across studies, family-centred approaches are not consistently defined as a single construct but are instead positioned in three overlapping ways: (i) as structured, evidence-based interventions such as psychoeducation and multifamily programmes; (ii) as a service delivery philosophy guiding organizational practice; and (iii) as an ethical or inclusive stance emphasizing collaboration with families. This conceptual variability influences how evidence is generated, interpreted, and translated into practice, and complicates direct comparison across studies.

Psychoeducation emerged as a key mechanism through which family-centred approaches are understood to operate. Studies describe that when families receive structured information about symptom patterns, medication regimens, and relapse warning signs, caregivers report greater confidence in supporting their relatives [51]. Authors frequently link enhanced caregiver understanding to earlier help-seeking, improved communication, and reduced family conflict [30,31]. While some of these claims are grounded in controlled intervention studies, others derive from qualitative or service-level reports. As such, psychoeducation should be understood as a widely endorsed and theoretically coherent component of family-centred care, rather than as a uniformly validated mechanism across all service contexts.

When conceptualized as an intervention, family-centred care is most strongly supported by controlled trials of structured programmes such as psychoeducation and multifamily interventions. These studies demonstrate improvements in relapse prevention, treatment adherence, and caregiver outcomes. However, this evidence applies specifically to manualized and structured interventions and should not automatically be generalized to all forms of family involvement described in the broader literature.

Implementation is shaped by cultural, structural, and systemic conditions. In settings where family interdependence is culturally normative, relatives are more likely to actively participate, whereas in contexts marked by stigma, families may withdraw to avoid social judgement [48]. Structural barriers including transport limitations, financial constraints, and rigid service schedules disproportionately affect low-income and rural families [35]. On the provider side, insufficient training, time pressures, and uncertainty regarding confidentiality constrain routine family inclusion [28,29]. These findings indicate that family-centred care is not achieved solely through clinical intention. Rather, it is contingent on organizational design, workforce preparation, and cultural responsiveness. Without systemic alignment, family involvement remains inconsistent and vulnerable to individual discretion.

When framed as a service philosophy, family-centred care is associated with improved engagement, continuity of care, and therapeutic alliance. However, the evidence supporting this perspective is largely observational or experiential, drawing on qualitative and cross-sectional studies rather than controlled evaluation. This suggests that while family involvement is widely valued in principle, its effectiveness depends heavily on service context and organizational capacity.

To illustrate how these challenges have been addressed within established intervention science, the McFarlane Multifamily Psychoeducation (MFPE) model provides a robust, evidence-based exemplar [7]. MFPE integrates psychoeducation, structured problem-solving, and peer support within a group-based format and has demonstrated effectiveness in reducing relapse and caregiver burden within specific diagnostic populations [7]. Within the context of this scoping review, MFPE is not presented as a universal solution but as an example of how conceptual clarity, structural design, and empirical evaluation can be aligned. Its relevance lies in demonstrating that family-centred care can be operationalized in ways that are both theoretically coherent and empirically testable.

As an ethical or inclusive practice, family-centred care reflects broader values of collaboration, respect, and shared decision-making. However, qualitative findings indicate that its enactment is uneven, shaped by stigma, cultural expectations, resource limitations, and professional boundaries. This highlights a gap between normative endorsement of inclusion and its practical realization in clinical settings.

The Stress–Vulnerability Model further helps to interpret findings related to relapse prevention, caregiver burden, and symptom management. Within this framework, family support functions as a buffering mechanism that reduces environmental stressors while enhancing coping resources, thereby lowering the likelihood of symptom exacerbation in vulnerable individuals. This aligns with evidence from the reviewed studies showing that structured family interventions are associated with improved relapse outcomes and reduced caregiver strain. Finally, Cognitive-Behavioural approaches offer an explanatory framework for understanding how psychoeducation and structured family interventions produce change. By improving caregiver knowledge, modifying maladaptive beliefs about mental illness, and enhancing problem-solving skills, family-centred interventions may influence both caregiver responses and patient behaviours. This helps explain the observed improvements in treatment adherence, communication, and reduced expressed emotion reported across intervention studies. Taken together, these theoretical perspectives provide a more coherent explanation of how and why family-centred approaches influence outcomes while also highlighting the multi-layered nature of implementation across clinical, relational, and systemic levels.

More broadly, this review highlights a critical tension in the literature: family-centred care is simultaneously treated as an evidence-based intervention, a service philosophy, and an ethical stance. Without conceptual precision, there is a risk that the strong evidence base of specific models is implicitly extended to broader, less substantiated practices. Addressing this ambiguity is essential for advancing both research and implementation. Family-centred care holds considerable promise. Realizing that promise, however, requires moving beyond rhetorical endorsement toward structurally supported, context-sensitive, and conceptually coherent practice. Future research should explicitly distinguish between these three conceptualizations and evaluate them separately, while service implementation and policy development should clearly specify which form of family-centred care is being adopted. This would strengthen both the precision of evidence interpretation and the effectiveness of real-world application.

### 4.1. Study Recommendations

A key recommendation is for mental health services to strengthen structured family involvement from intake through treatment to improve engagement and early detection of symptom changes. Improving clinician training in family-inclusive care, alongside creating dedicated family-support roles, can further enhance consistency. Finally, supporting caregiver wellbeing through family support groups and respite options, and conducting ongoing evaluation of family-centred interventions will help ensure that these approaches remain effective and sustainable.

### 4.2. Study Limitations

This review has several limitations that should be acknowledged. First, only studies published in English were included, which may have excluded relevant research conducted in non-English-speaking regions where family involvement in mental health care may differ significantly due to cultural and systemic factors. Second, the review was limited to literature published between 2000 and 2025, meaning that earlier foundational studies may not have been captured and recent but unpublished or in-press research may also have been missed. Third, the reviewed studies employed a wide range of methodologies including qualitative studies, cross-sectional surveys, observational designs, and a smaller number of controlled trials resulting in variability in study quality and limiting the ability to draw strong causal conclusions. The predominance of qualitative and descriptive studies, while valuable for understanding experiences, also restricts the generalizability of the findings. Additionally, methodological inconsistencies across studies, such as small sample sizes, non-random sampling, and limited longitudinal follow-up, further constrain the strength of the evidence. These limitations suggest caution in interpreting the results and highlight the need for more rigorous, diverse, and internationally inclusive research.

## 5. Conclusions

Family-centred approaches play a vital role in promoting inclusive mental health care for individuals with psychiatric disorders by enhancing treatment engagement, improving adherence, and supporting both service users and their caregivers. Psychoeducation and structured family involvement strengthen families’ knowledge, coping skills, and confidence, while culturally responsive practices and attention to structural barriers are critical for meaningful participation. Despite these benefits, implementation remains inconsistent due to challenges such as clinician training gaps, organizational limitations, and caregiver burden. Future research and practice should focus on expanding access, addressing systemic and cultural barriers, and evaluating long-term impacts to ensure that family-centred approaches are effective, sustainable, and adaptable across diverse mental health settings.

## Figures and Tables

**Table 1:** Eligibility criteria.

PCC Element	Inclusion Criteria	Exclusion Criteria
**Population**	Individuals are diagnosed with psychiatric disorders. Family members, caregivers, or significant others are involved in supporting individuals with psychiatric disorders.	Studies focusing on non-psychiatric populations. Studies involving only mental health professionals without family or patient perspectives.
**Concept**	Studies examining family-centred, family-focused, or family-inclusive approaches. Interventions such as family therapy, psychoeducation, family involvement programmes, Open Dialogue, and collaborative care. Policies, guidelines, models, or strategies that promote family inclusion in mental health care. Outcomes related to inclusivity, engagement, recovery, shared decision-making, or family wellbeing.	Studies discussing general caregiving without a family-centred mental health focus. Interventions unrelated to family involvement (e.g., purely pharmacological trials). Articles that describe family burden without linking it to a family-centred approach.
**Context**	Any mental health care setting (inpatient, outpatient, community, home-based, tele-mental health). Studies conducted in any cultural or geographic context.	Non-health care settings not linked to mental health service delivery (e.g., school-only programmes, correctional settings without clinical components).
**Time frame**	Publications from 2000–2025.	Studies published before 2000.
**Language**	English-language publications.	Non-English publications.
**Types of Evidence Sources**	Empirical studies: qualitative, quantitative, mixed methods.	Systematic reviews, scoping reviews, meta-analyses, editorials, letters to editors, opinion pieces lacking empirical or conceptual depth, conference abstracts without full data, and full text not accessible.

**Table 2:** Full search strategy.

Database	Search Strategy
PubMed	(“Family-Centred Care”[MeSH] OR “Family centred Approach OR “Family Involvement” OR “Family Support”) AND (“Mental Disorders”[MeSH] OR “Psychiatric Disorders” OR “Mental Illness” OR “Psychiatric Conditions”) AND (“Inclusive Education” OR “Inclusive Care” OR “Inclusive Mental Health”)
CINAHL	(MH “Family-centred Care”) OR (“family centred approach*” OR “family involvement” OR “family support”) AND (MH “Psychiatric Disorders” OR “mental disorders” OR “mental illness” OR “psychiatric conditions”) AND (“inclusive mental health” OR “inclusive care” OR “inclusion”)
PsycINFO	(MH “Family-centred Care” OR “family centred approach*” OR “family involvement” OR “family support”) AND (MH “Psychiatric Disorders” OR “mental disorders” OR “mental illness” OR “psychiatric conditions”) AND (“inclusive mental health” OR “inclusive care” OR “inclusion”)
Scopus	TITLE-ABS-KEY(“family-centred care” OR “family centred approach*” OR “family involvement” OR “family support”) AND TITLE-ABS-KEY(“psychiatric disorders” OR “mental disorders” OR “mental illness” OR “psychiatric conditions”) AND TITLE-ABS-KEY(“inclusive mental health” OR “inclusive care” OR “inclusion”)
Embase	‘family-centred care’/exp OR ‘family centred approach*’ OR ‘family involvement’ OR ‘family support’ AND ‘psychiatric disorder’/exp OR ‘mental disorder*’ OR ‘mental illness’ OR ‘psychiatric condition*’ AND ‘inclusive mental health’/exp OR ‘inclusive care’ OR ‘inclusion’
Web of Science	TS = (“family-centred care” OR “family centred approach*” OR “family involvement” OR “family support”) AND TS = (“psychiatric disorders” OR “mental disorders” OR “mental illness” OR “psychiatric conditions”) AND TS = (“inclusive mental health” OR “inclusive care” OR “inclusion”)

**Table 3:** Summary of thematic synthesis.

Stage	What Was Done	Evidence from Included Studies	Output
1. Data extraction (study findings)	Relevant findings on family involvement, psychoeducation, caregiving outcomes, and implementation factors were extracted from all included studies	All studies [25,26,27,28,29,30,31,32,33,34,35,36,37,38,39,40,41,42,43,44,45,46,47,48] contributed findings including engagement outcomes, caregiver burden, psychoeducation effects, cultural influences, and service barriers	Dataset of extracted findings
2. Initial coding (inductive line-by-line coding)	Extracted findings were coded inductively to identify meaningful units of information	Examples of codes: improved treatment adherence [31,32,33], psychoeducation improves caregiver confidence [32,33], stigma limits engagement [36,37,38], service barriers [39,40,41,42,43,44,45], caregiver burden [45,46,47,48]	Initial list of descriptive codes
3. Code grouping (categorisation)	Similar codes were grouped into broader categories through constant comparison across studies	Engagement-related codes [25,26,27,28,29,30,31,32,33]; psychoeducation codes [31,32,33]; cultural/stigma-related codes [34,35,36,37,38]; implementation barriers [39,40,41,42,43,44,45]; caregiver wellbeing codes [45,46,47,48]	Preliminary thematic categories
4. Theme development (pattern identification)	Categories were refined into higher-order themes based on recurring patterns across studies	Studies contributing to engagement [25,26,27,28,29,30,31,32,33]; psychoeducation [31,32,33]; cultural/structural influences [34,35,36,37,38]; barriers [39,40,41,42,43,44,45]; caregiver wellbeing [45,46,47,48]	Five preliminary themes
5. Theme refinement (ensuring distinctiveness)	Themes were reviewed to reduce overlap and ensure conceptual clarity across the dataset	Overlap resolved between engagement, psychoeducation, and family involvement; barriers separated from outcomes; caregiver wellbeing distinguished as a distinct outcome domain	Finalized thematic framework
6. Final thematic framework (reported findings)	Final themes were synthesized and presented in the Results section of the review	All included studies [25,26,27,28,29,30,31,32,33,34,35,36,37,38,39,40,41,42,43,44,45,46,47,48] mapped across one or more themes	Final five themes presented in manuscript

**Table 4:** Study characteristics.

Author, Year, and Title	Methodology	Setting (Country)	Population (n)	Intervention/Approach	Key Findings
[25] Szmukler et al., 2003. *An exploratory randomized controlled trial of a support programme for carers of patients with a psychosis.*	exploratory randomized controlled trial	Camberwell, South-East London	80	Family psychoeducation intervention	A carer support programme was feasible and acceptable and showed potential in reducing caregiver burden and distress while improving coping and perceived support.
[26] Treasure et al., 2001. *The experience of caregiving for severe mental illness: a comparison between anorexia nervosa and psychosis.*	Qualitative research	Southeast London	159	Family-focused therapy	Caregiving burden is high across both anorexia nervosa and psychosis, but differs in nature, with caregivers of individuals with anorexia experiencing more emotional strain and complexity in managing the illness.
[27] Heru (2000). *Family functioning, burden, and reward in the caregiving for chronic mental illness.*	Qualitative research	South Carolina	60	Family psychoeducation	Caregiving for chronic mental illness is associated with both significant burden and perceived rewards, with family functioning influencing the overall caregiving experience.
[28] Chen & Greenberg (2004). *A positive aspect of caregiving: The influence of social support on caregiving gains for family members of relatives with schizophrenia.*	Cross-sectional	Washington, DC	560	Family-inclusive approach	Social support is positively associated with caregiving gains, enhancing caregivers’ sense of personal growth and positive meaning in caring for relatives with schizophrenia.
[29] Tarricone et al., 2006. *The experience of carers of patients with severe mental illness: a comparison between London and Bologna.*	Cross-sectional	London and Bologna	168	Family-focused practice	Caregiver experiences of burden and support vary across contexts, with differences between London and Bologna reflecting the influence of mental health service systems and social support structures on caregiving.
[30] Glanville & Dixon (2005). *Caregiver burden, family treatment approaches and service use in families of patients with schizophrenia.*	Qualitative research	USA	50	Family psychoeducation	Higher caregiver burden is associated with greater service use, and that family treatment approaches can influence both caregiver strain and engagement with mental health services.
[31] Addington et al., 2005. *Three-year outcome of family work in an early psychosis program.*	Cross-sectional	Canada	185	Family psychoeducation	Family work in early psychosis programmes is associated with improved long-term outcomes, including reduced relapses and better patient and family functioning over three years.
[32] Bäuml et al., 2006. *Psychoeducation: a basic psychotherapeutic intervention for patients with schizophrenia and their families. Schizophrenia bulletin.*	Randomized control	Munich	81	Family psychoeducation	Psychoeducation for patients with schizophrenia and their families improves knowledge, enhances treatment adherence, and contributes to relapse prevention.
[33] Miklowitz et al., 2000. *Family-focused treatment of bipolar disorder: 1-year effects of a psychoeducational program in conjunction with pharmacotherapy.*	randomized controlled design	Boulder/Denver, Colorado	101	Family-focused psychoeducational	Family-focused psychoeducational treatment combined with pharmacotherapy improves treatment adherence and reduces relapse rates in individuals with bipolar disorder over one year.
[34] Ran et al., 2016. *Family caregivers and outcome of people with schizophrenia in rural China: 14-year follow-up study.*	Prospective design	China	510	Family-inclusive approach	Sustained family caregiving is associated with improved long-term outcomes, including better symptom management and survival among individuals with schizophrenia over a 14-year period.
[35] Peng et al., 2019. *Disease-related stressors of caregiving burden among different types of family caregivers of persons with schizophrenia in rural China.*	Retrospective design	China	300	Family-inclusive approach	Caregiving burden among family caregivers of individuals with schizophrenia is driven by disease-related stressors, with variations in burden depending on caregiver type and role.
[36] Deng et al., 2023. *Quality of life among family caregivers of people with schizophrenia in rural China.*	Cross-sectional	China	269	Family-inclusive approach	Family caregivers of individuals with schizophrenia experience reduced quality of life, which is significantly influenced by caregiving burden, social support, and economic and emotional stressors.
[37] Wang et al., 2023. *Affiliate stigma and caregiving burden among family caregivers of persons with schizophrenia in rural China.*	Cross-sectional	China	253	Family-inclusive approach	The study revealed the higher caregiving burden among family caregivers of individuals with schizophrenia.
[38] Ran et al., 2022. *Effectiveness of enhancing contact model on reducing stigma of mental illness among family caregivers of persons with schizophrenia in rural China: A cluster randomized controlled trial.*	cluster randomized controlled trial	China	231	Family-inclusive approach	Enhanced contact intervention was effective in reducing stigma among family caregivers of individuals with schizophrenia, while also improving attitudes and understanding of mental illness.
[39] Reupert et al., 2015. *The family-focused practice of primary care clinicians: a case of missed opportunities.*	Mixed method	Australia	21	Family-focused practice	Missed the opportunity to involve the family members due to limited resources.
[40] Cree et al., 2015. *Carers’ experiences of involvement in care planning: a qualitative exploration of the facilitators and barriers to engagement with mental health services.*	Qualitative research	UK	14	Family involvement approach	Carers’ involvement in care planning is shaped by both facilitators within mental health services.
[41] Wonders et al., 2019. *Family inclusion in mental health service planning and delivery: Consumers’ perspectives.*	Qualitative research	South Africa	13	Family involvement approach	This study found that consumers view family inclusion in mental health service planning and delivery as beneficial for recovery.
[42] Small et al., 2017. *Understanding experiences of and preferences for service user and carer involvement in physical health care discussions within mental health care planning.*	Qualitative research	UK	47	Family involvement approach	Both service users and carers prefer meaningful involvement in physical health care discussions within mental health care planning, but actual involvement is often limited by communication gaps and service constraints.
[43] Kokanović et al., 2018. *Supported decision-making from the perspectives of mental health service users, family members supporting them and mental health practitioners.*	Qualitative research	Australia	92	Family-focused psychoeducational	Supported decision-making is valued by service users, family members, and practitioners, but its implementation is shaped by tensions between autonomy, clinical authority, and the practical role of family involvement in care decisions.
[44] Schout et al., 2017. *The use of family group conferences in mental health: Barriers for implementation.*	Qualitative research	Dutch	60	interviews	This study found that the implementation of family group conferences in mental health care is hindered by organizational, professional, and attitudinal barriers, including limited resources, resistance to change, and unclear role definitions among stakeholders.
[45] Chien et al., 2016. *The effectiveness of manual-guided, problem-solving-based self-learning programme for family caregivers of people with recent-onset psychosis: a randomized controlled trial with 6-month follow-up.*	A randomized controlled trial	Hong Kong	116	Family-involved practice	Manual-guided, problem-solving self-learning programme for family caregivers of individuals with recent-onset psychosis significantly improved caregiver coping, reduced burden, and enhanced patient outcomes over a 6-month follow-up period.
[46] Chien et al., 2013. *The effectiveness of mutual support group intervention for Chinese families of people with schizophrenia: a randomised controlled trial with 24-month follow-up.*	A randomized controlled trial	Hong Kong	35	Family involvement practice	Mutual support group interventions for families of individuals with schizophrenia significantly improve caregiver’s wellbeing, reduce burden, and enhance long-term patient and family outcomes over a 24-month follow-up period.
[47] Chien et al., 2016. *A randomized controlled trial of clinician-supported problem-solving bibliotherapy for family caregivers of people with first-episode psychosis.*	A randomized controlled trial	Hong Kong	116	Family involvement practice	Caregivers’ coping skills reduces caregiving burden and distress and supports better patient and family outcomes in first-episode psychosis.
[48] Miklowitz et al., 2008. *Family-focused treatment for adolescents with bipolar disorder: results of a 2-year randomized trial.*	randomized controlled trial	USA	58	Family involvement practice	Family-focused treatment for adolescents with bipolar disorder leads to improved mood stability, reduced relapse rates, and better family functioning over a 2-year period.

## Data Availability

No new data were created or analyzed in this study. Data sharing is not applicable to this research.

## Supplementary Materials

The following supporting information can be downloaded at: https://www.mdpi.com/article/10.3390/ijerph23080952/s1, Table S1: Preferred Reporting Items for Systematic reviews and Meta-Analyses extension for Scoping Reviews (PRISMA-ScR) Checklist.
